# CherryZZZ: A Protocol for a Randomized, Double-Blind, Placebo-Controlled, Cross-Over Pilot Study Testing Tart Cherry Juice in Older Adults with Self-Reported Insomnia

**DOI:** 10.3390/nu18060922

**Published:** 2026-03-14

**Authors:** Esther VanderMark, Amir Baniassadi, Alex Wolfe, Dennis P. Cladis, Alyssa B. Dufour, Courtney L. Millar

**Affiliations:** 1 Hinda and Arthur Marcus Institute for Aging Research, Hebrew SeniorLife, Boston, MA 02131, USA; esthervandermark@hsl.harvard.edu (E.V.); amirbaniassadi@hsl.harvard.edu (A.B.); alexwolfe@hsl.harvard.edu (A.W.); alyssadufour@hsl.harvard.edu (A.B.D.); 2 Department of Medicine, Beth Israel Deaconess Medical Center, Harvard Medical School, Boston, MA 02215, USA; 3 Department of Food Science and Technology, Virginia Polytechnic Institute and State University, Blacksburg, VA 24061, USA; dcladis@vt.edu; 4 Department of Human Nutrition, Foods, and Exercise, Virginia Polytechnic Institute and State University, Blacksburg, VA 24061, USA

**Keywords:** flavonoids, sleep, aging, inflammation, melatonin, tart cherry juice

## Abstract

**Introduction:** Two small, preliminary pilot studies report that 2 weeks of daily tart cherry juice consumption (half of the dose in the morning, half of the dose at night) may increase sleep quantity (assessed via a sleep diary or 1 night of polysomnography) in older adults with insomnia. A study of longer duration, with doses closer to bedtime, and daily objective monitoring of sleep via a wearable device may potentiate the observed impact of tart cherry juice intake on sleep. With the proposed changes to the study protocol, it is paramount to evaluate the study’s feasibility. **Methods:** The current study is a single-site, randomized, double-blind, cross-over pilot study in 20 older adults with self-reported insomnia. Eligible individuals will be randomly assigned to consume 16 oz. of tart cherry juice/day or placebo juice for 4 weeks each, separated by a 3-week washout period. Information on study feasibility, including recruitment rate, retention rate, safety, compliance, and study practicality, will be collected, as well as pre- and post-arm evaluations of sleep quantity/quality and biomarkers related to melatonin, cortisol, serotonin, and inflammation. **Discussion:** Identification of a dietary intervention that improves sleep quantity and quality may serve as a novel and feasible approach for older adults who suffer from insomnia. If successful, such a strategy would help mitigate the plethora of health consequences associated with poor sleep.

## 1. Introduction

An alarming 40–70% of older adults are estimated to have chronic sleep problems, which consequently increases the risk of detrimental health outcomes, including physical ailments, mental health issues, and cognitive decline [1]. Effective pharmacological strategies are often paired with severe adverse effects, which dissuade older adults from seeking treatment and leave them suffering from poor sleep [2]. Thus, there is an urgent need to identify safe, non-pharmacological strategies that optimize sleep to support healthy aging in older adults and to minimize adverse outcomes that result from poor sleep.

Diet offers a safe, non-pharmacological strategy to modulate sleep. There is a growing interest in functional foods and their role in contributing to overall health. Functional foods are defined as foods containing bioactive components with the capability to provide additional health benefits beyond their basic nutritional value [3]. These additional health benefits include, but are not limited to, reducing inflammation, supporting physiological functions, and improving metabolic efficiency [3]. In fact, tart cherry juice may be a functional food for improved sleep. Two small pilot studies in older adults with insomnia (i.e., a condition marked by poor sleep) reported that intake of tart cherry juice for 2 weeks increased sleep quantity (i.e., total sleep time), but not quality (i.e., patient-reported satisfaction with sleep) [4,5]. Poor sleep quality is associated with mortality and other diseases of aging [6,7,8,9], which underscores the importance of improving both sleep quantity and quality. Interventions targeting improved sleep quality typically have durations of at least 4 weeks, as previously reviewed [10]. Given that the previous studies had 2-week durations of tart cherry juice intake, longer studies may be required to see increases in both sleep quantity and quality.

Additionally, the previous studies asked participants to consume half of their tart cherry juice dose in the morning and the other half at night. If tart cherry juice theoretically does impact sleep, it may make more sense to administer both doses closer to the onset of sleep. With a larger intake of liquid closer to bedtime, the risk of nocturia increases, especially for older adults. A final change to the previous studies we considered was the monitoring of sleep quantity. The previous studies utilized a sleep diary or a 1-night polysomnographic sleep study to monitor sleep quantity [4,5]. Unfortunately, sleep diaries are subject to recall bias and sleep quantity is subject to a high degree of variation. Thus, an objective assessment of sleep quantity via a wearable actigraphy device that captures sleep quantity nightly in the comfort of a participant’s home may provide a more precise estimate of the impact of tart cherry juice on sleep.

Several questions arise with the proposed protocol changes that need answering before launching an efficacy trial. Will participants comply with a longer study duration? Will administering both doses closer to bedtime induce nocturia and disrupt sleep? Will older adults be willing to utilize a wearable device that monitors sleep each night, and how much missing data will there be? Therefore, it is critical to address the feasibility of our revised protocol to answer the questions above.

**Figure 1:**
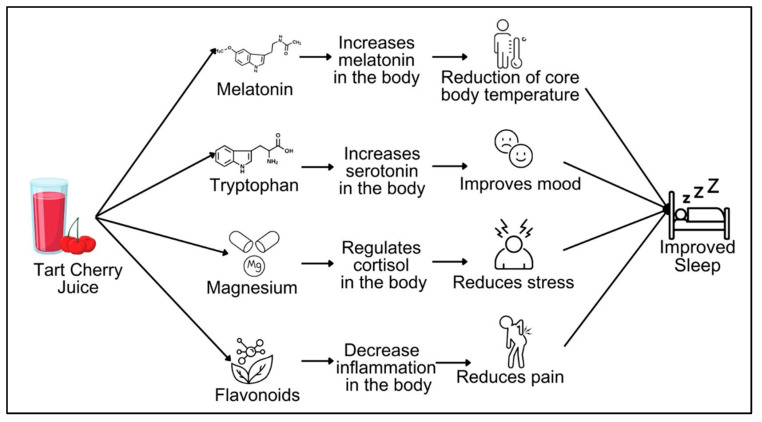
Theoretical model of the impact of tart cherry juice and pathways relevant to sleep. Tart cherry juice provides several compounds that hypothetically modulate specific biomarkers, which then have subsequent effects on sleep-relevant factors.

Another critical benefit of feasibility studies is the generation of preliminary data on proposed outcomes. Despite preliminary trials, the mechanisms that may be responsible for the impact of tart cherry juice on sleep remain unclear. Tart cherry juice is made from Montmorency cherries, which contain melatonin, an important compound involved with the initiation of sleep [11]. Additionally, there are other nutritional and bioactive compounds found in tart cherries, some of which may be relevant for sleep [12,13,14,15,16], including: tryptophan, a precursor to the mood hormone serotonin [16,17,18,19]; magnesium, a micronutrient that helps regulate the production of the stress hormone cortisol [20,21]; and flavonoids, plant-based compounds that protect cells from damage and lower inflammation in the body [12,14]. It is the synergy between these specific compounds that we hypothesize is the mechanism for how tart cherry juice may impact sleep (Figure 1). Tart cherry-derived melatonin, tryptophan, magnesium, and flavonoids have a down-stream effect on biomarkers. These biomarkers then have a subsequent effect on sleep-related factors (e.g., reduction in core body temperature [11,22,23], improved mood [19,24,25,26], reduced stress [27,28,29], and reduced pain [30,31,32]). Thus, we also proposed to evaluate potential mechanisms that may underly the impact of tart cherry juice on sleep to identify pathways to explore in future studies.

The objective of this study is to determine the feasibility of our revised tart cherry juice intervention protocol (called CherryZZZ) in older adults, and generate preliminary data on outcomes and mechanisms related to sleep. We hypothesize that recruitment for and implementation of a 4-week tart cherry juice intervention in older adults with self-reported insomnia are feasible. We further hypothesize that intake of tart cherry juice will increase sleep quantity (via a wearable actigraphy device), sleep quality (validated via a questionnaire, the Pittsburgh Sleep Quality Index), urinary melatonin, and serum serotonin related markers, as well as decrease urinary cortisol and serum inflammatory markers.

## 2. Materials and Methods

### 2.1. Study Design and Regulatory Approvals of the Study

The aim of this study is to determine the feasibility of our revised protocol and gather preliminary data on sleep-related outcomes and biomarkers after tart cherry juice intake in older adults with self-reported insomnia. Our study is called CherryZZZ and is an individual-level, double-blind, randomized, cross-over, pilot study in older adults with self-reported insomnia. The study was approved by the Advarra IRB (#Pro00078791). The protocol that is described in this paper is Version 3, dated 31 January 2025. The study was registered at Clinicaltrials.gov (identifier: NCT06786494) on 1 October 2025, where the protocol and statistical analysis can be accessed.

### 2.2. Study Population, Recruitment, and Randomization

Our target population is older adults aged ≥65 years with self-reported insomnia. We intend to enroll 20 individuals in our study. The eligibility criteria are listed in Table 1. Additionally, individuals could be excluded for any other reason/condition if the PI and investigative team believe this intervention would be unsafe. In 2025, individuals in the greater Boston area were recruited via local newspaper and internet advertisements, physician referrals, registries of research volunteers, presentations at senior housing sites in the greater Boston area, and use of an online recruitment company. All interested individuals will be asked to provide verbal consent to complete an initial eligibility screen during a phone conversation with study personnel. Potentially eligible participants will then schedule an in-person screening visit, where the informed consent form will be reviewed with study staff. Written informed consent will be obtained by study personnel at the beginning of the in-person screening visit. Each participant will be given a unique study identification number and data will not include any of the participant’s protected health information (PHI). All participant-identifying information will be stored and managed on a password-protected secured database server.

Our trial proposes to enroll 20 individuals. In an attempt to account for the impact of treatment order, we will randomly assign individuals to the order in which they receive the intervention and placebo. That is, some individuals will start with the intervention and then the placebo; others will start with the placebo and then the intervention. This process will be carried out while maintaining participant and investigator blinding. Block randomization (sequence generated via SAS version 9.4) will be employed to reduce the impact of season/time. For every block of 4 participants, 2 will be randomized to the tart cherry juice and the remaining 2 will be randomized to the placebo juice.

### 2.3. Study Intervention, Beverages, Blinding, and Compliance

Screened and eligible participants will be asked to avoid cherry-containing foods/beverages and supplemental forms of flavonoids, magnesium, and tryptophan for the entire 12-week study duration. They will also be asked to keep a sleep diary by recording the time they got into bed and the time they got out of bed and wear an actigraphy watch to objectively monitor sleep quantity each night of the entire study. Prior to their baseline visit, participants will be asked to complete a one-week duration of sleep quantity assessment via the wearable actigraphy watch and fill out a 3-day diet record. Then participants will come to their baseline visit and undergo study outcome assessments and provide the requested biological samples (blood/urine/fecal). Fasted blood will be collected in the morning hours to avoid the impact of the circadian rhythm on any markers. Then participants will be randomized to the order in which they consume tart cherry juice or the placebo beverage (of similar calories, color, and taste) for 4 weeks. Participants will be asked to consume half of their dose (i.e., 8 fluid ounces) at around 5 pm and the remaining half (i.e., 8 fluid ounces) about an hour before bed. After 4 weeks of the first arm, the participants will perform follow-up outcome assessments and provide urine/blood/fecal samples. After a 3-week washout period, individuals will be asked to repeat the same visits with the other arm of the intervention.

Tart cherry juice will be purchased from a commercial supplier (Shoreline Fruit^TM^), which will be responsible for preparing and bottling the tart cherry juice. Frozen or fresh tart cherries will be juiced and bottled according to the manufacturer’s standard protocols. The main nutritional and bioactive components in an 8-fluid-ounce serving of tart cherry juice are described in Table 2. In addition to the sleep-related components already mentioned, tart cherry juice also provides iron and potassium, whose contents are higher in tart cherries when compared to other fruits. The intervention beverages will be prepared following proper food safety/handling protocols and bottled. The placebo beverage will be an unsweetened black cherry powder (i.e., Kool-Aid^TM^) that is matched to the caloric and sugar content of tart cherry juice. The placebo beverage will be prepared by the Rutgers Food Innovation Center following good manufacturing practices. The placebo beverage will have a similar taste and color to tart cherry juice, but will theoretically not contain tart cherries or their bioactive compounds (i.e., magnesium, melatonin, tryptophan, or flavonoids).

The placebo will be bottled in the same bottle type as the tart cherry juice; however, it will be marked with a letter, number, or symbol that differs from the one used to identify the tart cherry juice. The key that decodes which letter/number corresponds with which product will be kept secure by an independent party. Thus, the participants, study staff, and analyzers of the data will be blinded to the letter/number that is used to identify the tart cherry juice vs. the placebo. At the end of the data collection and data analysis, the study team will be unblinded.

Individuals will be provided with an allotment of the intervention products, and additional bottles will be delivered by our study staff as needed. Compliance will be evaluated by daily compliance logs and quantity of empty and full bottles of juice. The logs will request information about how much was consumed and the timing of the doses. At the study visits, participants will be asked to return all bottles of juice to estimate the number of intended doses that are consumed.

### 2.4. Study Measurements

Table 3 outlines the assessments that will be performed at each visit. In the beginning of the study, an assessment of an individual’s medical history, health behaviors, existing/previous medical conditions, current medications, smoking status, use of alcohol, etc., will be performed to characterize the participants. They will also be asked about their subjective perception of social support, and connectedness will be evaluated using a validated questionnaire [38]. Throughout the intervention, participants will record 3-day diet records (consisting of 2 weekdays and 1 weekend day) to evaluate dietary intake over the course of the study. Diet records will be entered into the dietary analysis program, the Nutrition Data System for Research, to estimate dietary intake of nutrients. Information on relevant symptoms, including gastrointestinal distress, appetite, pain, etc., is collected by self-report at each study visit. Information on sleep-related factors, including use of alcohol/caffeine, anxiety, and emotional affect, will also be evaluated by validated questionnaires. Participant vital signs (i.e., body temperature, pulse, and seated blood pressure), height, and weight will be measured at each in-person visit.

Participants will also provide biological samples at each in-person visit. Up to 16 mL of fasting blood will be collected by a trained phlebotomist using sterile procedures. Blood is centrifuged at 3.6 × 1000 RPM for 15 min at 4 °C for EDTA plasma or after 30 min of clotting at room temperature at 3.6 × 1000 RPM for 10 min at 4 °C for serum. Aliquots of serum/plasma are then stored at −80 °C for future analyses of planned biomarkers. Approximately 30 mL of spot urine will be collected, processed, and stored at −80 °C for future analyses. We will aim to collect urine in the morning hours between 8 and 10 am to avoid the influence of the circadian rhythm on cortisol. Batch analyses of planned biomarkers will be measured by a reputable lab.

Participants will also have the option to partake in two optional study assessments that are intended for further investigations of potential mechanisms relevant to sleep, including analysis of phenolic metabolites in urine and analysis of the gut microbiota in fecal samples. If participants are willing, they will be asked to provide a 24 h urine collection instead of the planned spot urine collection. This requires individuals to collect all of their urine for the 24 h preceding select study visits using a provided urine container. Urine will be acidified to a final concentration of 0.1% formic acid and stored at −80 °C until analysis. Regardless of whether participants opt for the spot or the 24 h urine sample, batch analyses of planned urinary biomarkers (i.e., cortisol and 6-sulphatoxymelatonin) will be measured by a reputable lab. Additionally, if participants are willing, they will be asked to collect at least 1 (no more than 3) separate fecal sample before select visits. Samples will be collected as is in a sterile collection tube, without any preservation medium, that will be provided to participants. There will be a collection for select visits, as indicated in Table 3. Participants will temporarily store the self-collected fecal samples in their own freezer (−17 °C) until study staff transports them to our lab at Hebrew SeniorLife for sample processing and long-term storage. The fecal samples are planned to be used for whole genome sequencing and analysis of the gut microbiota.

### 2.5. Primary, Secondary, and Exploratory Outcomes

Our primary outcomes are aspects of study feasibility.

1. Recruitment Rate will be evaluated as the number of individuals that need to be screened to be enrolled that are randomized to the intervention.
2. Attrition will be evaluated as the number of individuals who are randomized that withdraw from the intervention in medias res.
3. Compliance with Dose will be evaluated as the number of intended doses consumed. The number of doses consumed will be estimated through the self-reported compliance logs and returned juice bottles.
4. Ability to Collect Data will be evaluated as the percentage of missing data points of sleep quantity from the wearable Garmin watch that will monitor sleep each night.
5. Practicality will be evaluated as the self-reported willingness to continue the dietary regimen after study completion.

Secondary endpoints include sleep quantity and quality, as well as sleep-related biomarkers (i.e., melatonin, serotonin, cortisol, and inflammatory markers). Exploratory outcomes are evaluations of mood, stress and pain.

1. Sleep Quantity and Quality: Sleep quantity will be assessed subjectively and objectively. For the subjective measure, participants will be given a daily log or journal to document aspects of their sleep, including time of sleep onset, time of rising, number of disturbances, and self-rated satisfaction with sleep. Participants are asked to complete this log every day they are in the study. To objectively measure sleep quantity, participants are asked to wear a digital device that measures sleep quantity. Participants will be asked to wear the device throughout the intervention to objectively capture quantitative aspects of sleep (e.g., time in bed, number of times awake). Quality of sleep will be evaluated using the validated Pittsburgh Sleep Quality Index (PSQI) self-report questionnaire [39].
2. Sleep-related Biomarkers (Melatonin, Serotonin, and Cortisol): Melatonin will be estimated by measuring the major urinary metabolite of melatonin, 6-sulphatoxymelatonin. Serotonin will be estimated by measuring the kynurenine/tryptophan ratio in the blood. Cortisol will be estimated by measuring cortisol in the blood. Serum C-reactive protein and interleukin-6 will be measured as markers of inflammation. All markers will utilize commercially available quantitative immunoassays.
3. Exploratory Outcomes (Mood, Stress, and Pain): Mood, stress, and pain will be evaluated by validated self-report questionnaires [40,41,42].

### 2.6. Participant Safety and Data Management

All participants will be tracked from the time of enrollment through to their study completion or withdrawal. All events will be logged using forms modeled after the forms that are provided by the National Institute of Aging Clinical Research Toolbox. For this specific study, AEs will be primarily captured using a self-report symptoms questionnaire where participants disclose a symptom and the associated severity level. A participant’s disclosure of any symptom that is moderate or severe will qualify as an AE unless the symptom and a similar severity level are disclosed at baseline. A serious adverse event (SAE) is defined as any experience that results in death, a life-threatening situation, or hospitalization. Each AE will be graded by the PI for severity, relatedness, and expectedness. All AEs/SAEs will be reviewed by the PI and reported to the IRB as appropriate. All other adverse events/study incidents that do not require immediate reporting to the IRB will be logged and reported to the IRB following the appropriate reporting times as defined by the Advarra IRB. Any AE that (1) is unexpected in nature, severity, or frequency, (2) is possibly, probably, or definitely related, and (3) suggests that the research places participants at a greater risk of harm than previously known or recognized will be reported to the appropriate regulatory boards within 2 weeks of the AE.

Since this is a single-site, phase 1 pilot study, without high risk, our study will not require an official Data and Safety Monitoring Board or Safety Officer. However, to ensure and monitor participant safety, the study will comply with all safety reporting required by the IRB and adhere to the following participant stopping rule. Any participant experience of a severe adverse event will be subject to PI review to determine their continuation in the study. Any report of a serious adverse event (SAE) that is categorized as directly related to the study will result in the participant’s discontinuation from the study.

### 2.7. Sample Size, Statistical Power, and Analysis

All analyses will be performed via intent to treat and further evaluated per protocol. As a first step, we will assess distribution characteristics of the primary and secondary outcomes. Where appropriate, transformation of variables to combat skew or other irregularities will be employed. Participant characteristics will be summarized using means, medians, standard deviations, interquartile regions and ranges for continuous variables and sample counts and proportions generated for discrete characteristics. Comparability of treatment arms will be assessed on potentially confounding characteristics using tabular and graphical methods. Due to the preliminary nature of this pilot study, no formal power analysis was performed. Sensitivity analyses that exclude participants with mean compliance below 80% will be explored in a per-protocol analysis. Other potential sensitivity analyses include excluding those who have missing sleep data or stratifying by baseline fiber consumption, levels of depressive symptoms, or levels of sedentary behavior, etc.

## 3. Discussion

The aim of our study is to determine the feasibility of our newly revised tart cherry juice protocol that is intended to improve sleep in older adults with self-reported insomnia. Optimal sleep is an essential part of healthy aging. When sleep is disturbed, especially in older adults, it increases the risk of physical morbidity, mental health disorders, and cognitive decline [1]. Two previous pilot studies provide promising preliminary results suggesting that intake of tart cherry juice appears to improve sleep.

In 2010, Pigeon et al. [5] published a small randomized, double-blind, cross-over pilot study. They report that 2-week intake of tart cherry juice (8 fl oz in the morning and 8 fl oz in the evening) reduced the time spent awake after sleep onset compared to a placebo beverage in 15 older adults with self-reported insomnia. While promising, the aspects of sleep quantity were captured via self-reported sleep diaries, which are subject to significant recall bias for sleep measures. Losso et al. [4] followed with a study published in 2019 that had a very similar study design, duration, and dosing pattern. In eight older adults with self-reported insomnia, tart cherry juice consumption increased total sleep time by approximately 80 min based on a one-night polysomnography test compared to placebo. While polysomnography overcomes the recall bias that is associated with self-reported sleep diaries, the use of one night of sleep measurement is limiting. Furthermore, participants are not in their usual sleep environment and the test may not reflect their usual sleep pattern. Therefore, there may be improvements to the study protocol that may help increase the potency of the effect of tart cherry juice.

The previous studies were of relatively short duration (2 weeks), required the consumption of half of the tart cherry juice dose in the morning (which may not be physiologically relevant to sleep), and captured sleep either by self-reports or with only one night of polysomnography. To address these limitations, we made several changes to the previous studies’ protocols, including extending the duration of intake to 4 weeks, adjusting the timing of both doses to be closer to the onset of sleep, and implementing the use of a wearable device and daily sleep diaries to capture sleep quantity each night for the entire study duration.

Introduction of the changes described above to the previous protocol may impact study feasibility. For example, the longer dose duration may impact adherence to the intervention, changing the timing of juice doses to be closer to bedtime may induce nocturia, and the use of a wearable device may impact willingness and ability to collect data. Therefore, it is critical to assess feasibility outcomes—including recruitment rate, attrition, compliance, ability to collect data, and practicality—to determine if the revised protocol is a practical, safe, and useful intervention to help promote sleep in older adults.

In addition to modifying the protocol, our study builds upon the results from the previous two pilot studies by continuing to explore potential mechanisms. Previous work suggests that the melatonin content of tart cherry juice and the flavonoid component may be underlying the potential benefits for sleep [4,5]. However, there are other compounds such as magnesium and tryptophan in tart cherries that may influence sleep via changes in biomarkers and clinical outcomes (some of which are described in Figure 1). Thus, our study provides a more comprehensive assessment of biomarkers and clinical outcomes that may be impacted by tart cherry juice. However, a limitation of our study is that we are studying an entire beverage, and not the specific nutrients/bioactive compounds. Therefore, we are unable to discern the impact of other nutrients that are provided by tart cherry juice, like iron and potassium, on sleep.

Our study is limited by other factors as well. Primarily, our sample size is still relatively small. This was intentional since our main goal for this study was not to evaluate the clinical efficacy of tart cherry juice for sleep, but rather to evaluate the feasibility of our newly revised protocol. With such a small sample size, we are likely going to have limited generalizability based on who is recruited for the study. Furthermore, with our protocol changes, older adults may not be willing to comply with a longer study duration, have negative experiences of nocturia due to the beverages being consumed closer to bedtime, and not want to monitor their sleep with a wearable watch for the entire duration of our study. However, these limitations and questions are exactly why we are determining the feasibility of these protocol changes before implementing a large-scale efficacy trial.

Findings on compliance, safety, and practicality will inform the feasibility of implementing a tart cherry juice intervention in a large-scale trial that may strengthen findings and, in turn, impact the implementation of effective non-pharmacological sleep strategies in real-world settings. Specifically, results from this protocol will shed light on older adults’ willingness and ability to continue drinking tart cherry juice on their own, affirming this dietary approach as a feasible and sustainable strategy. Furthermore, we plan to capture data on potential mechanisms linking tart cherry juice and sleep in older adults suffering from insomnia. Clarification of these mechanisms will identify pathways to explore in future studies, as well as optimize practical and biologically relevant treatment duration and dose timing. If our study protocol is proven as a feasible strategy, this evidence will support and motivate a large-scale clinical efficacy trial to evaluate the impact of tart cherry juice intake on sleep. As a result, we will be positioned to potentially identify a safe, widely accessible and non-pharmacological strategy of functional foods to help mitigate chronic sleep problems in older adults. Doing so will allow the ever-growing older adult population to obtain adequate and quality sleep, which is essential to healthy aging.

## Figures and Tables

**Table 1:** Eligibility criteria.

Inclusion Criteria
Age ≥ 65 years
Self-reported insomnia *
Usual bedtime between 8 pm and 1 am
**Exclusion Criteria**
Unwilling to follow the study protocol
Unable to properly use the wearable device
Self-reporting a diagnosis of sleep disorders other than insomnia (e.g., sleep apnea)
Reporting current and consistent use of sleep aids or hypnotic prescriptions (e.g., trazadone) **
Self-reporting cognitive impairment, dementia, or another neurological disorder
On unstable medications (i.e., changed within the last 3 months) for other conditions
Allergic to the intervention products
Self-reported history of diabetes
Current alcohol or drug use disorder ***
Are excessive caffeine drinkers (≥ 5 cups of caffeinated beverages a day)

* Defined as trouble sleeping 3 or more nights a week for at least 6 months and/or an Insomnia Severity Index score ≥ 10 points [33]. ** If individuals were willing to avoid use of supplemental melatonin at least 2 weeks before starting the study and throughout the study, participants were considered eligible. *** Alcohol disorder was defined by the Alcohol Use Disorders Identification Test as a score ≥ 4 points [34,35], while drug use disorder was defined as a Drug Abuse Screening Test score > 2 points [36,37].

**Table 2:** Nutritional information for an 8 oz serving of tart cherry juice.

Nutritional Component	Amount
Calories	140 kcal
Sodium	30 mg
Total Carbohydrates	33 g
Total Sugar	25 g
Protein	1 g
Calcium	12 mg
Iron	1 mg
Potassium	386 mg
**Nutritional/Bioactive Component Related to Sleep**	
Melatonin	0.135 ug
Magnesium	24 mg
Tryptophan	20 mg
Flavonoids	115 mg *

* Based on USDA Database for Flavonoid Content of Selected Foods Release 3.3.

**Table 3:** Study assessments at each visit.

Visit	1	2	3	4	-	5	6	7
	Week 0	Week 1	Week 3	Week 5	Week 7	Week 8	Week 10	Week 12
**Planned Study Assessments**								
Screening	X							
Medical History	X							
Health Behaviors and Social Network	X							
Relevant Symptoms		X	X	X		X	X	X
Vitals		X		X		X		X
Height	X							
Weight	X	X		X		X		X
Sleep Quality		X		X		X		X
Sleep Quantity	X	X	X	X	X	X	X	X
Related Sleep Factors			X	X			X	X
Blood and Spot Urine Samples		X		X		X		X
Depressive Symptoms		X	X	X		X	X	X
Stress		X	X	X		X	X	X
Pain		X	X	X		X	X	X
3-Day Diet Record	X			X	X			X
Compliance				X		X		X
**Optional Study Assessments**								
24-Hour Urine Collection *	X			X	X			X
Fecal Sample	X			X	X			X

* If participants opt for the optional 24 h urine assessment, they will not provide a spot urine sample.

## Data Availability

The data presented in this study are available on request from the corresponding author due to the requirement of proper regulatory documents and approvals.
